# Possible Role of Arginase-1 in Concomitant Tumor Immunity

**DOI:** 10.1371/journal.pone.0091370

**Published:** 2014-03-10

**Authors:** Michael J. Korrer, John M. Routes

**Affiliations:** 1 Department of Pediatrics, Medical College of Wisconsin, Milwaukee, Wisconsin, United States of America; 2 Department of Microbiology and Molecular Genetics, Medical College of Wisconsin, Milwaukee, Wisconsin, United States of America; 3 Children's Research Institute, Milwaukee, Wisconsin, United States of America; 4 Cancer Center, Medical College of Wisconsin, Milwaukee, Wisconsin, United States of America; University of North Carolina at Greensboro, United States of America

## Abstract

The expression of Adenovirus serotype 2 or serotype 5 (Ad2/5) E1A in tumor cells reduces their tumorigenicity *in vivo* by enhancing the NK cell mediated and T cell mediated anti-tumor immune response, an activity that correlates with the ability of E1A to bind p300. We determined if E1A could be used as a molecular adjuvant to enhance antigen-specific T cell responses to a model tumor antigen, ovalbumin (OVA). To achieve this goal, we stably expressed a fusion protein of E1A and OVA (MCA-205-E1A-OVA), OVA (MCA-205-OVA) or a mutant version of E1A unable to bind p300 and OVA (E1A-Δp300-OVA) in the B6-derived, highly tumorigenic MCA-205 tumor cell line. MCA-205-E1A-OVA tumor cells were over 10,000 fold less tumorigenic than MCA-205-OVA, MCA-205-E1A-Δp300-OVA, or MCA-205 in B6 mice. However, immunization of B6 mice with live MCA-205-OVA, MCA-205-E1A-Δp300-OVA and MCA-E1A-OVA tumor cells induced nearly equivalent OVA-specific CD4 T cells and CD8 CTL responses. Further studies revealed that mice with primary, enlarging MCA-205-OVA or MCA-205-E1A-Δp300-OVA tumors on one flank exhibited OVA-specific anti-tumor T cell responses that rejected a tumorigenic dose of MCA-205-OVA cells on the contralateral flank (concomitant tumor immunity). Next we found that tumor associated macrophages (TAMs) in progressive MCA-205-OVA tumors, but not MCA-205-E1A-OVA tumors that expressed high levels of arginase-1, which is known to have local immunosuppressive activities. In summary, immunization of mice with MCA-205 cells expressing OVA, E1A-Δp300-OVA or E1A-OVA induced equivalent OVA-specific CD4 and CD8 anti-tumor responses. TAMs found in MCA-205-OVA, but not MCA-205-E1A-OVA, tumors expressed high levels of arginase-1. We hypothesize that the production of arginase-1 by TAMs in MCA-205-OVA or MCA-205-E1A-Δp300-OVA tumor cells leads to an ineffective anti-tumor immune response in the tumor microenvironment, but does not result in inhibition of a systemic anti-tumor immunity.

## Introduction

Expression of the Adenovirus E1A oncoprotein in primary cells results in cellular immortalization [1]. Cells stably expressing E1A and the helper protein E1B have been shown to be oncogenic in immunosuppressed rodents [2], [3]. Paradoxically, in rodent models the expression of Adenovirus serotype 2 or serotype 5 (Ad2/5) E1A in tumor cell lines significantly reduces tumorigenicity [4] (we now refer to Ad2/5 E1A as simply E1A). The ability of E1A to reduce tumorigenicity is dependent on the induction of a robust NK cell and T cell anti-tumor immune response [5] and correlates with the ability of E1A to bind the transcriptional co-adaptor molecule p300 or CBP [6]. p300 and CBP are highly homologous co-activators of transcription with intrinsic histone-acetyl transferase activity and will hereafter be referred to as simply p300 [7]. The expression of E1A, but not mutant forms of E1A that do not bind p300 (E1A- Δp300), also upregulates NKG2D ligands [8] and sensitizes cells to lysis by macrophages, NK cells and immune effector molecules utilized by these cells [9]–[13].

Based on these anti-tumorigenic activities of E1A, we sought to determine if E1A could be used to enhance antigen specific, anti-tumor T cell responses to MCA-205 tumor cells that express a model tumor antigen, ovalbumin (OVA). MCA-205 tumor cells that expressed a fusion protein of E1A and OVA elicited an effective anti-tumor T cell response and were rendered non-tumorigenic. Surprisingly, immunization of mice with live MCA-205-OVA or MCA-205-E1A-Δp300-OVA tumor cells elicited a robust anti-tumor immune response, despite forming progressive tumors at the primary site of immunization (concomitant tumor immunity). Further studies examined a possible mechanism whereby immunization of B6 mice with MCA-205-OVA or MCA-205-E1A-Δp300-OVA could induce systemic anti-tumor immunity but fail to clear a local tumor burden.

## Materials and Methods

### Mice

Inbred C57BL6/J (B6), B6.129S7-Rag1tm1Mom/J (RAG^−/−^), B6.SJL-Ptprca Pepcb/BoyJ (CD45.1), C57BL/6-Tg(TcraTcrb)1100Mjb/J (OT-I), and B6.Cg-Tg(TcraTcrb)425Cbn/J (OT-II) mice were purchased from The Jackson laboratories (Bar Harbor, ME). OT-I mice express a transgene for a T cell receptor that recognizes ovalbumin (OVA) residues 257–264 in the context of H-2K^b^ [14]. OT-II mice express a transgene for a T cell receptor that recognizes chicken OVA residues 323–339 in the context of I-A^b^ [15]. Male mice six to nine weeks in age were used. All animal work was reviewed and approved by the Medical College of Wisconsin Institutional Animal Care and Use Committee.

### Reagents

Roswell Park Memorial Institute (RPMI) medium with 5% Fetal Bovine Serum (FBS) (RPMI-5) or 10% FBS (RPMI-10) supplemented with Glutamax (Invitrogen, Carlsbad, CA), glucose and antibiotics was used for all cell culture. FBS (Atlanta Biologicals, Flowery Branch, GA) was heat inactivated for 45 minutes at 56°C. OVA_257–264_ peptide was purchased from Sigma.

### Flow cytometry

Flow cytometry was performed with a LSR II (BD biosciences, San Jose, CA) using BD FACSDiva software. Flow cytometry analysis was performed using Flow Jo software (Tree Star, Ashland, OR). Antibodies specific to mouse CD3ε (145-2C11) Alexa Fluor 488 (AF-488); Fluorescein (FITC), CD8a (5H-10) PE; Pacific Orange (PO), CD45.1 (A20) Allophycocyanin (APC), NK1.1 (PK136) PE, and GR-1 (Rb6-8C5) APC were purchased from Biolegend (San Diego, CA). Antibodies specific to mouse CD3ε (145-2C11) AF-780, CD4 (GK1.5) Efluor 450 (EF-450); Peridinin Chlorophyll (PerCP), CD11b (M170) EF-450, CD11c (N418) PerCP, F4/80 (BM8) APC-Cy7, CD45 (30-F11) PE, H-2K^b^ OVA_257–264_ complex (25-D1.16) APC were purchased from Ebiosciences (San Diego, CA).

### Cloning strategy

The wild-type Adenovirus 5 *E1A* gene was cloned from Adenovirus 5 (GenBank ID: AY147066.1) bp 44–596,713–1029 Forward primer: 5′-CGT ACT GAA TTC TAA GGT ACC ATG GGC TCC ATC GGT GCA GC-3′, Reverse primer: 5′-GCT GCA CCG ATG GAG CCT GGC CTG GGG CGT TTA CAG CT-3′ by PCR. A mutant version of E1A unable to bind p300 (E1A-Δp300) was cloned from the Adenovirus E1A mutant *dl1104* using the same primers as E1A. E1A-Δp300 has a deletion in amino acids 48–60, which eliminates p300 binding [16]. The OVA gene was cloned from the pAC-OVA plasmid (GenBank ID: J00895.1), coding sequence (CDS) Forward primer: 5′-CGT ACT GAA TTC TAA GGT ACC ATG GGC TCC ATC GGT GCA GC-3′, Reverse primer: 5′-CGT ACT CTC GAG TTA AGG GGA AAC ACA TCT GCC-3′ by PCR. The resulting OVA gene expresses a slightly truncated OVA protein from AA 3–383 that included the relevant H-2K^b^ and I-A^b^ epitopes. The truncated version of OVA was made, as we found it could readily be expressed in MCA-205 cells, whereas, we could not express full-length OVA_1–386_ in MCA-205 cells (data not shown). The respective genes were cloned into pLXSN using XHOI and EcoRI restriction sites. The E1A-OVA and E1A-Δp300-OVA chimeric genes were generated through overlapping PCR using the following primers: E1A forward and reverse primer (listed above) plus the E1A-OVA primer 5′-AGC TGT AAA CGC CCC AGG CCA GGC TCC ATC GGT GCA GC-3′ and OVA reverse: 5′-CGT ACT CTC GAG TTA AGG GGA AAC ACA TCT-3′. The chimeric gene was inserted into pLXSN using EcoRI and Xho-I (New England Biolabs, Ipswich, MA) restriction sites. DNA sequencing by the Human and Molecular Genetics Sequencing core at the Medical College of Wisconsin verified the fidelity of OVA, E1A-OVA, and E1A-Δp300-OVA genes.

### Cell lines and cloning

MCA-205, a C57BL/6 derived fibrosarcoma cell line, was kindly provided by N. Restifo (National Cancer Institute, National Institutes of Health, Bethesda, MD) [17]. MCA-205 lines that stably expressed E1A-OVA (MCA-205-E1A-OVA), E1A-Δp300-OVA (MCA-205-E1A-Δp300-OVA), or OVA (MCA-205-OVA) were generated by transfection of MCA-205 cells with pLXSN-E1A-OVA, pLXSN-OVA, or pLXSN-E1A-Δp300-OVA using the Amaxa basic nucleofector kit for primary mammalian fibroblasts (Lonza, Basel, Switzerland) and a Nucleofector II (Lonza, Basel, Switzerland). Cell selection was done in media containing 1 mg/mL G418 (Sigma-Aldrich, St Louis, Missouri). Resistant colonies were screened for E1A expression and OVA expression by western blot analysis.

### Immunoprecipitation/Western blot detection of OVA

Lysates were generated from tumor lines from 10×10^6^ cells in 1 mL of radioimmune precipitation buffer (RIPA) consisting of 50 mM Tris-HCl; pH 7.4, 150 mM NaCl; 1 mM EDTA; 1% Triton X-100; 0.5% sodium deoxycholate; 0.1% sodium dodecyl sulfate (SDS); and 1 mM phenylmethylsulfonyl fluoride (PMSF) (Sigma-Aldrich, St Louis, Missouri). OVA was immunoprecipitated with rabbit polyclonal anti-OVA antibody (ab1221) (Abcam, Cambridge, England). The samples were analyzed by Western blot analysis. E1A bands were detected with mouse anti-E1A mAB m73 hybridoma supernatant (produced locally) and OVA bands were detected using mouse anti-OVA mAb (1E7) (Abcam, Cambridge, England). Protein bands were visualized using the Odyssey Infrared Imager (LI-COR Biosciences, Lincoln, Nebraska). Protein bands were quantified using Image Studio software (LI-COR Biosciences, Lincoln, Nebraska).

### Tumor induction studies

Quantitative tumor induction studies were performed as previously described [8]. Briefly, mice were administered serial log dilutions of tumor cells subcutaneously (s.c.) in the flank and monitored for tumor growth twice weekly using digital calipers. Animals were euthanized by CO_2_ followed by cervical dislocation when tumors reached 20 mm in diameter, if tumors ulcerated, or at the end of a 12-week monitoring period. Tumor producing dose 50 (TPD_50_) values, which are the log_10_ of the number of cells required to form tumors, were calculated using the Spearman-Karber Formula.

### Quantification of OVA specific CD8 and CD4 T cells

OT-I transgenic CD8 T cells were harvested from the spleens of OT-I^+^RAG^−/−^CD45.1^+^ mice and OT-II transgenic CD4 T cells were harvested from the spleens of OT-II^+^RAG^−/−^CD45.1^+^ mice. 1×10^5^ CD45.1^+^ OT-I CD8 T cells or 1×10^6^ CD45.1^+^ OT-II CD4 T cells were administered i.v. via retro-orbital injection into B6 mice. The following day, mice were administered 1×10^5^ live MCA-205-OVA, MCA-205-E1A-OVA or MCA-205-E1A-Δp300-OVA cells s.c. in the hock (the lateral tarsal region just above the ankle). Five days (OT-I) or nine days (OT-II) following tumor injection the popliteal lymph nodes were removed and the CD45.1^+^ OT-I or OT-II T cells were quantitated by flow cytometry by staining for CD45.1^+^ CD3^+^ CD8^+^ T cells or CD45.1^+^ CD3^+^ CD4^+^ T cells, respectively. The absolute number of cells was determined by multiplying the percentage of the target cell population by the total number of cells in the lymph node.

### *In vivo* CTL assay

B6 mice were primed with 1×10^6^ live tumor cells s.c. in the flank. Seven days later an *in vivo* CTL assay was performed on the primed mice [18]. B6 splenocytes pulsed with OVA _257–264_ peptide were used as targets. OVA pulsed splenocytes were labeled with a low dose (1 μM) of CFSE for one minute in 5% FBS PBS. Untreated splenocytes were labeled with a high dose (10 μM) CFSE for one minute in 5% FBS PBS. The two CFSE labeled target splenocytes groups were mixed equally and injected i.v. into primed mice. 10×10^6^ total target cells were administered to the mice. Four hours later, spleens were removed and splenocytes were analyzed by flow cytometry for CFSE expression. The ratio of OVA pulsed splenocytes (low CFSE) to unpulsed splenocytes (high CFSE) was used to determine specific killing. Specific killing was calculated as follows:

Specific lysis =  1- R_Naive_/R_exp_ X 100; R =  % OVA pulsed/% non-pulsed.

### Concomitant tumor induction studies

Mice were administered three injections of 1×10^5^ live tumor cells s.c. in the flank five days apart in 100 μL of PBS. Five days after the final injection (day 20) the mice were challenged with serial log dilutions of MCA-205-OVA cells on the contralateral flank and the TPD_50_ was determined. For some studies CD3 T cells were depleted on days 17, 19, 22 and 24 by i.p. injection of 10 μg anti-CD 3ε mAb 2C11 (BioXcell, West Lebanon, NH) or control Hamster IgG (BioXcell, West Lebanon, NH) into primed mice as previously described [19]. Mice were challenged with 1×10^4^ MCA-205-OVA cells on the contralateral flank and the percentage of mice tumor free from the MCA-205-OVA challenge was determined.

### Immune infiltrate of MCA-205-OVA tumors

B6 mice were administered 1×10^6^ live MCA-205-OVA cells s.c. in the flank. When the tumor reached a diameter of 10 mm (∼ two weeks) tumors were excised. Tumors were minced with scissors and digested with collagenase Type I 5 U/mL (Sigma-Aldrich, St Louis, Missouri), Deoxyribonuclease I 50 U/mL (Sigma-Aldrich, St Louis, Missouri), and Hyaluronidase, Type II 5 U/mL (Sigma-Aldrich, St Louis, Missouri) in 10 mL of RPMI-5 at 37°C for two hours. The digestion was stopped by the addition of 5 mL of 10 mM EDTA and incubated at 37°C for 15 minutes. A single cell suspension was generated by crushing the tissue with glass slides and passing through 40 μm filters. Cells were then characterized by flow cytometry.

### Tumor associated macrophage arginase expression

Tumors were excised from mice and a single cell suspension was generated as described above. Macrophages were purified using FACS and lysed in.4% Triton-X lysis buffer at a concentration of 1×10^5^ cells per 100 µL of lysis buffer. Arginase expression was quantified using the QuantiChrom Arginase Assay kit (Bioassay systems, Hayward, CA), following manufacturer's directions.

### Statistics

Statistical differences between groups were calculated using the ANOVA and Dunnett multiple comparison tests. Mann-Whitney tests were used to compare two sets of data. Survival curves were compared using the log-rank (Mantel Cox) test; p values less than.05 were considered significant. Unless otherwise stated, all data are presented as mean ± standard error of mean (SEM). Statistical analysis was done using Prism version 5.0a software (GraphPad Software, La Jolla, CA). (*, p<0.05; **, p<0.01; ***, p<0.001).

## Results

### Expression of E1A-OVA, OVA or E1A-Δp300-OVA in MCA-205 cells

**Figure 1:**
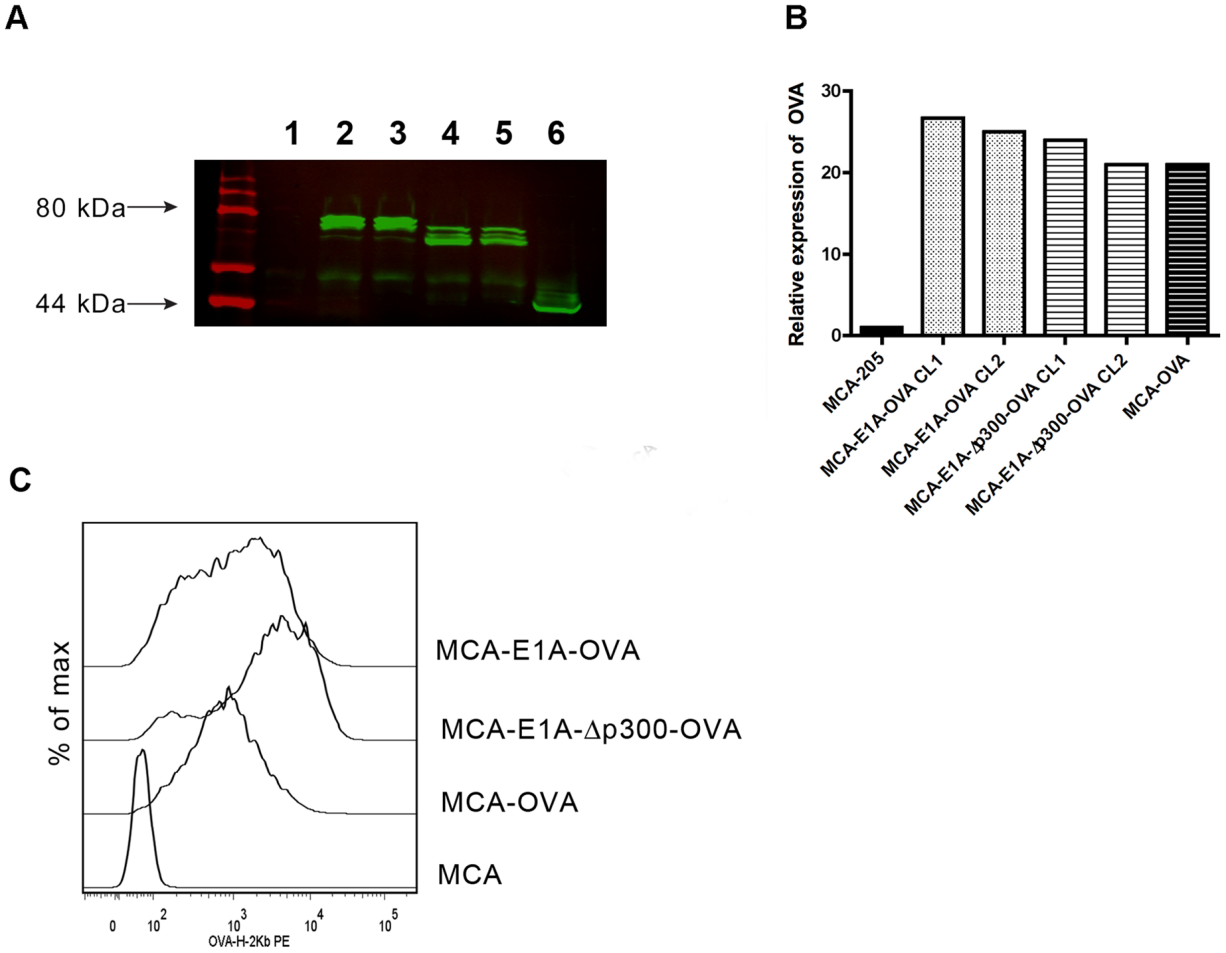
MCA-205 transfectants express OVA, E1A-Δp300-OVA, or E1A-OVA. **A**) Cell lysates were immunoprecipitated for OVA followed by Western blotting for OVA. Western blot analysis of OVA expression in the MCA-205 (lane 1), MCA-205-E1A-OVA Clone 1 (CL1) (lane 2), MCA-205-E1A-OVA CL2 (lane 3), MCA-205-E1A-Δp300-OVA CL1 (lane 4), MCA-205-E1A-Δp300-OVA CL2 (lane 5) and MCA-OVA (lane 6) lines. **B**) The relative density of the OVA-specific band was quantitated relative to MCA-205. **C**) Expression of OVA_257–264_ peptide bound to H-2K^b^ was determined by flow cytometry for MCA-205, MCA-205-OVA, MCA-205-E1A-Δp300-OVA and MCA-205-E1A-OVA using the mAb, 25-D1.16, which is specific for the OVA_257–264_ peptide bound to H-2K^b^. A histogram comparing relative expression levels of OVA_257–264_ peptide in the context of H-2K^b^ is shown. Data shown is one representative experiment of three independent experiments.

In an effort to determine if E1A could augment anti-tumor-specific T cell responses to OVA, we first generated MCA-205 tumor lines (see Materials and Methods) that expressed a slightly truncated OVA _3–383_ (MCA-205-OVA), a fusion protein of full length E1A and OVA_3–383_ (MCA-205-E1A-OVA) or a fusion protein of mutant E1A lacking amino acids 48–60, which results in the inability of E1A to bind p300 (E1A-Δp300) and OVA_3–383_ (MCA-205-E1A-Δp300-OVA). All MCA-205-OVA, MCA-205-E1A-Δp300-OVA and MCA-205-E1A-OVA tumor cells all expressed readily detectable amounts of OVA (Figure 1 A), although the amount of OVA was slightly higher in the MCA-205-E1A-OVA tumor line (Figure 1 B). OVA was detected at the expected molecular weight of approximately 43 kDa, whereas E1A-OVA and E1A-Δp300-OVA bands were detected at significantly higher molecular weights due to the addition of the E1A or E1A-Δp300 proteins.

Next, we confirmed that the OVA protein was processed and presented by the class I molecule H-2K^b^ by staining the various cell lines with the 25-D1.16 antibody which recognizes OVA_257–264_ peptide in the context of H-2K^b^. MCA-205-OVA, MCA-205-E1A-Δp300-OVA and MCA-205-E1A-OVA tumor cells expressed large amounts of OVA_257–264_ H-2K^b^ complexes on the cell surface (Figure 1 C). These data demonstrate that MCA-205 lines were generated that stably express E1A and/or OVA and that the OVA protein is processed and presented on the cell surface.

### Tumorigenicities of MCA-205-OVA, MCA-205-E1A-Δp300-OVA and MCA-205-E1A-OVA cells in B6 mice

**Figure 2:**
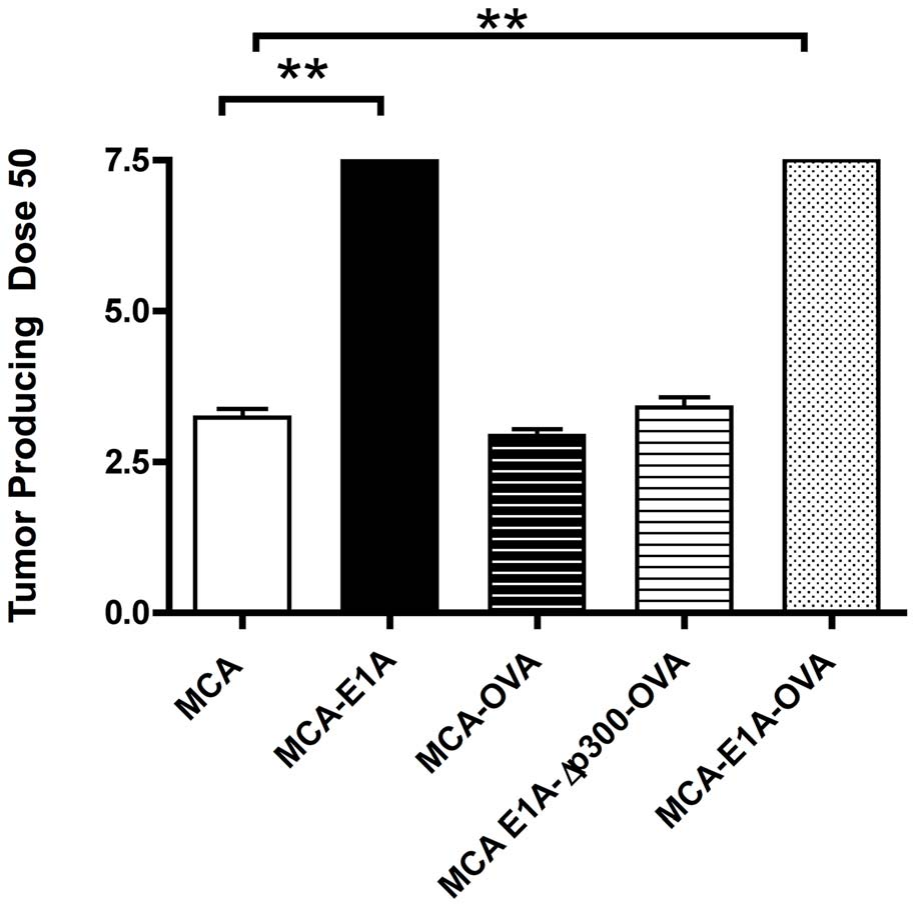
Stable expression of E1A-OVA reduced tumorigenicity of MCA-205 tumor cells. B6 mice were injected s.c. in the flank with serial log dilutions of MCA-205, MCA-205-OVA and MCA-205-E1A-Δp300-OVA tumor cells from 1×10^2^–1×10^5^ cells; and MCA-205-E1A and MCA-205-E1A-OVA tumor cells from 1×10^5^–1×10^7^ cells. The TPD_50_ (log_10_ of the number of cells required to form tumors in 50% of the mice) was calculated 12 weeks later. MCA-205-E1A and MCA-205-E1A-OVA cells failed to form tumors at the highest dose, 1×10^7^. Data shown is the mean ± SEM of three experiments with three mice at each tumor dose per experiment. **, p<0.01.

We next determined if E1A-OVA expression in MCA-205 tumor cells retained E1A *in vivo* biological activity by measuring the tumorigenicity of MCA-205, MCA-205-E1A, MCA-205-OVA, MCA-205-E1A-Δp300-OVA and MCA-205-E1A-OVA tumor lines (Figure 2). MCA-205-E1A has been previously shown to have substantially reduced tumorigenicity compared to MCA-205 cells [8] and served as a positive control. Tumorigenicity was measured by determining the tumor producing dose 50 (TPD_50_), which is the log_10_ of the number of tumor cells required to form tumors in half of the B6 mice (Material and Methods). MCA-205-E1A and MCA-205-E1A-OVA tumor cells were non-tumorigenic at the highest challenge dose (1×10^7^ cells) and were at least 10,000 fold less tumorigenic than MCA-205, MCA-205-OVA or MCA-205-E1A-Δp300-OVA tumor lines. MCA-205-OVA and MCA-205-E1A-Δp300-OVA tumor cells were equivalently tumorigenic as MCA-205 tumor cells, indicating that expression of either OVA or E1A-Δp300-OVA in MCA-205 cells does not alter the intrinsic tumorigenicity of the MCA-205 line. In summary, these tumor induction studies showed that the E1A-OVA fusion protein retains the anti-tumorigenic activity of the E1A protein.

### Characterization of T cell responses to MCA-205-OVA and MCA-205-E1A-OVA cells

**Figure 3:**
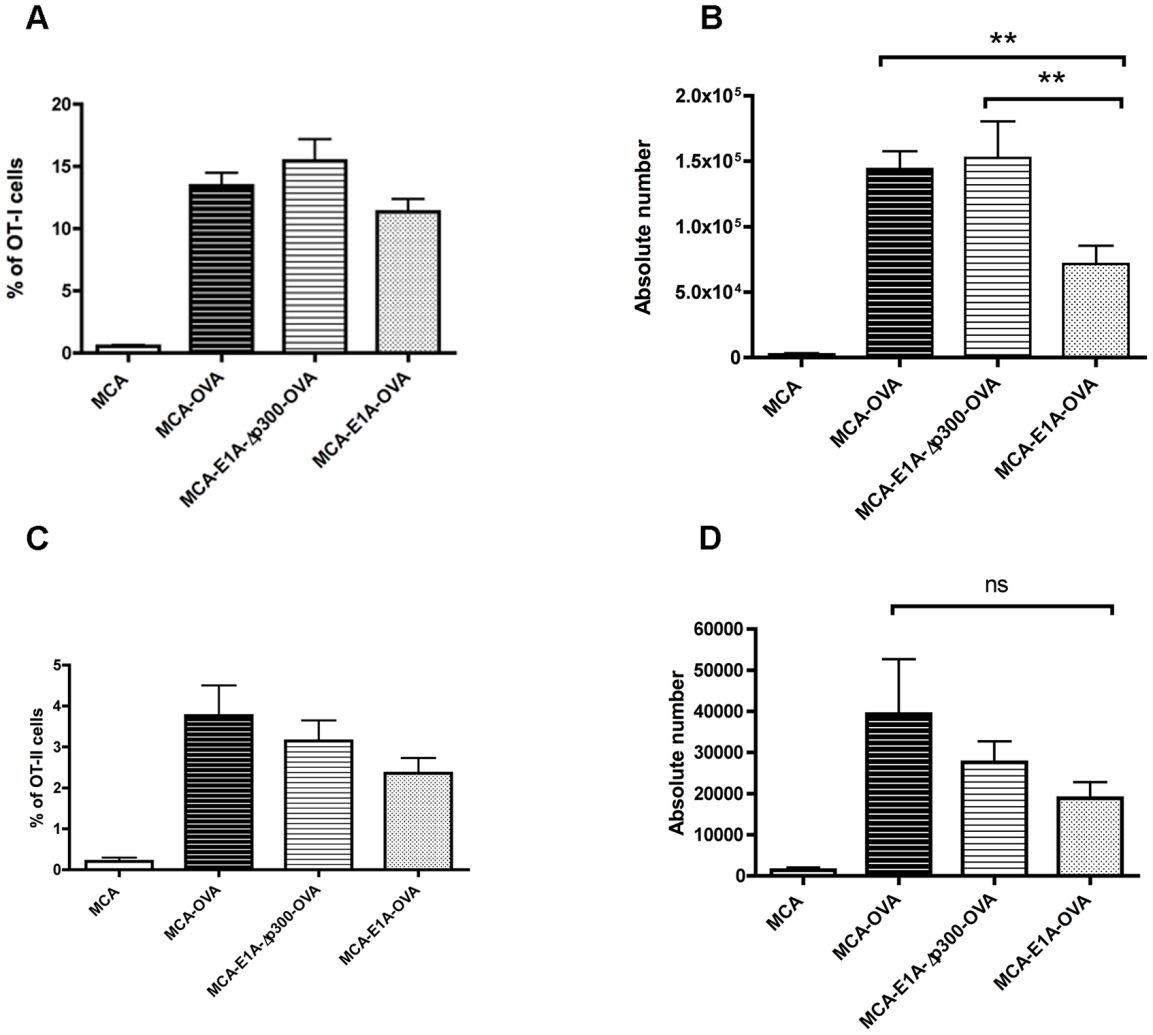
Immunization with MCA-205-OVA, MCA-205-E1A-Δp300-OVA and MCA-205-E1A-OVA cells expand similar numbers of OVA-specific T cells *in vivo*. **A, B**) B6 mice were injected with 1×10^5^ CD45.1^+^ OT-I cells and then 1×10^5^ MCA-205, MCA-205-OVA, MCA-205-E1A-Δp300-OVA, or MCA-205-E1A-OVA tumor cells 24 hours later s.c. into the hock. Five days later the draining lymph nodes were removed and the number of OT-I cells was determined by flow cytometry analyzing for CD45.1^+^CD3^+^CD8^+^ cells. Data shown is the percent of OT-I cells of the CD8 T cell population (**A**), and the absolute number of OT-I cells in draining lymph node (**B**). Data shown in **A, B** is the mean ± SEM, from three experiments with 6–13 mice per group. **C, D**) Experiments were performed as in **A, B**, except 1×10^6^ CD45.1^+^ OT-II cells were administered, and the draining lymph nodes were collected on day nine. The number of OT-II CD4 T cells was determined by flow cytometry by analyzing for CD45.1^+^CD3^+^CD4^+^ cells. Data shown is mean ± SEM, from two experiments with 3–8 mice per group. Data were analyzed by ANOVA followed by Tukey's HSD post hoc analyses. *, p<.05; **, p<.01; ns, no significance.

As previously noted, MCA-205-OVA and MCA-205-E1A-Δp300-OVA tumor cells were found to be highly tumorigenic whereas MCA-205-E1A-OVA were non-tumorigenic at the highest challenge dose (Figure 2). One explanation for these results was that, in contrast to MCA-205-E1A-OVA cells, MCA-205-OVA or MCA-205-E1A-Δp300-OVA failed to induce a productive OVA–specific anti-tumor immune response *in vivo*. As an initial test of this hypothesis, we challenged B6 mice with the various live OVA expressing tumor lines and compared their ability to expand OVA specific CD4 T cells (OT-II cells) or CD8 T cells (OT-1 T cells) *in vivo*. 1×10^5^ CD45.1^+^ OT-I CD8 T cells isolated from CD45.1^+^OT-I^+^Rag^−/−^ mice and were adoptively transferred into CD45.2^+^ B6 mice. 24 hours later, MCA-205, MCA-205-OVA, MCA-205-E1A-OVA or MCA-205-E1A-Δp300-OVA cells were injected into the hock. Five days after tumor injection, the popliteal lymph nodes were removed and the number of OT-I cells was determined by enumerating the number of CD45.1^+^ CD8 T cells by flow cytometry (Figure 3 A, B). In contrast to MCA-205 cells, challenge of mice with MCA-205-OVA, MCA-205-E1A-Δp300-OVA or MCA-205-E1A-OVA tumor cells resulted in a significant expansion of OT-I cells in the draining lymph node (Figure 3 A, B). Although there was no significant difference in the percentage of OT-I cells in the popliteal lymph node following challenge with MCA-205-OVA, MCA-205-E1A-Δp300-OVA or MCA-205-E1A-OVA tumor cells (Figure 3 A), MCA-205-OVA or MCA-205-E1A-Δp300-OVA tumor cells had a significantly higher number of OT-I cells compared to mice challenged with MCA-205-E1A-OVA tumor cells (Figure 3 B). From these data, we concluded that mice challenged with tumorigenic MCA-205-OVA and MCA-205-E1A-Δp300-OVA tumor cells had a numerically more robust OVA-specific CD8 T cell response than mice challenged with the non-tumorigenic MCA-205-E1A-OVA tumor cells.

Next, we examined the anti-OVA CD4 T cell response to injection of MCA-205-OVA, MCA-205-E1A-Δp300-OVA, and MCA-205-E1A-OVA tumor lines in mice. 1×10^6^ CD45.1^+^OT-II CD4 T cells isolated from CD45.1^+^OT-II^+^RAG^−/−^ mice were adoptively transferred into CD45.2^+^ B6 mice and 24 hours later mice were challenged with MCA-205, MCA-205-OVA, MCA-205-E1A-Δp300-OVA, and MCA-205-E1A-OVA tumor lines. Nine days after tumor cell injection, the popliteal lymph nodes were removed and OT-II T cells were enumerated by flow cytometry. In contrast to MCA-205 cells, challenge of mice with MCA-205-OVA, MCA-205-E1A-Δp300-OVA or MCA-205-E1A-OVA resulted in an equivalent expansion of OT-II cells present in the draining lymph node (Figure 3 B, C) Collectively these data indicate that challenge of mice with MCA-205-OVA, MCA-205-E1A-Δp300-OVA or MCA-205-E1A-OVA tumor cells induced an OVA-specific CD4 and CD8 T cell response.

### Characterization of the functional T cell response to MCA-205-OVA and MCA-205-E1A-OVA cells

**Figure 4:**
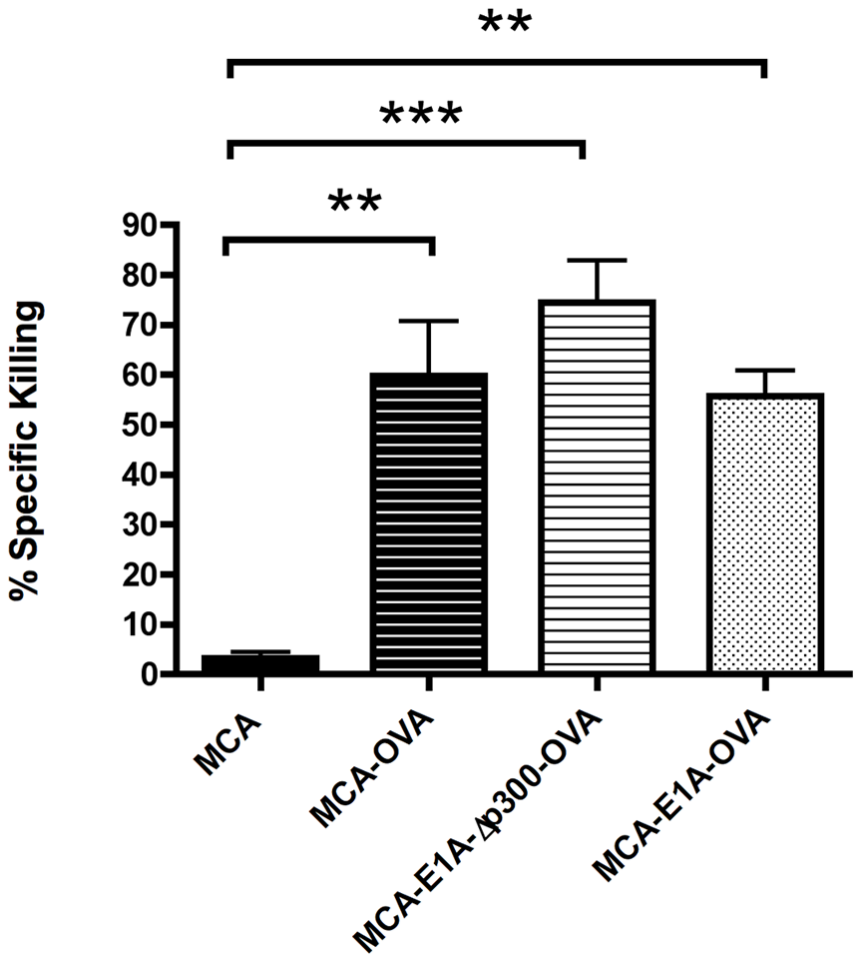
MCA-205-OVA, MCA-205-E1A-Δp300-OVA and MCA-205-E1A-OVA tumor cells elicited similar *in vivo* OVA-specific cytotoxic T cell responses. **A**) Mice were injected with 1×10^6^ live MCA-205, MCA-205-OVA, MCA-205-E1A-Δp300-OVA, or MCA-205-E1A-OVA tumor cells in the flank s.c. and an *in vivo* CTL assay against OVA pulsed splenocytes was performed seven days later. Data shown is the mean ± SEM from three experiments, with six mice per group. Data were analyzed by ANOVA followed by Tukey's HSD post hoc analyses. **, p<.01; ***, p<.001.

Challenge with tumorigenic MCA-205-OVA or MCA-205-E1A-Δp300-OVA cells expanded OVA-specific CD8 T cells slightly better than challenge with MCA-205-E1A-OVA cells. Therefore, we determined if there was a functional difference between the CD8 T cell responses in mice challenged with MCA-205-E1A-OVA or MCA-205-OVA tumor cells, which could account for differences in tumorigenicity. To test the functional response of OVA-specific CD8 T cells in mice to challenge with live MCA-205-OVA, MCA-205-E1A-Δp300-OVA or MCA-205-E1A-OVA cells, we performed an *in vivo* CTL killing assay (Figure 4). As expected, mice challenged with MCA-205 cells in the absence of OVA did not induce OVA-specific CTLs. In contrast, mice primed with MCA-205-OVA, MCA-205-E1A-Δp300-OVA or MCA-205-E1A-OVA cells induced equivalent OVA-specific CTL killing (Figure 4). These results suggest that injection of live MCA-205-OVA, MCA-205-E1A-Δp300-OVA or MCA-205-E1A-OVA cells elicits OVA-specific CTL capable of killing OVA expressing targets.

### Presence of Protective Systemic Tumor Immunity in Mice with Progressive MCA-205-OVA and MCA-205-E1A-Δp300-OVA Tumors

**Figure 5:**
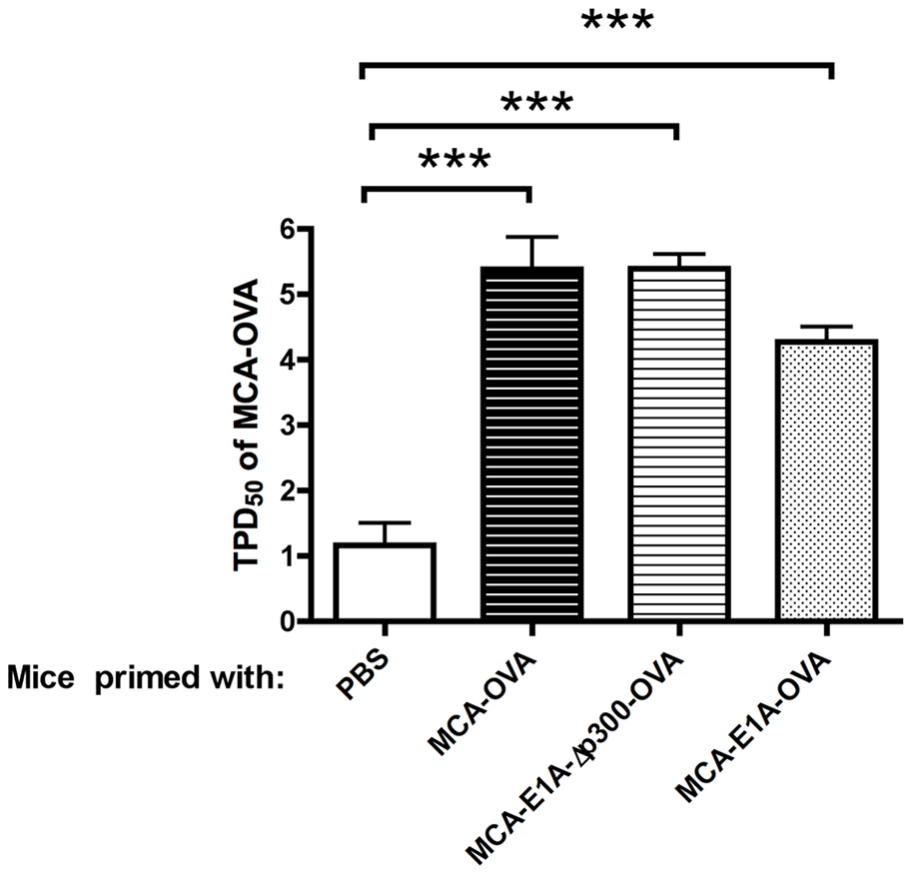
Immunization with MCA-205-OVA, MCA-205-E1A-Δp300-OVA and MCA-205-E1A-OVA tumor cells provided concomitant tumor immunity against MCA-205-OVA challenge on the contralateral flank of B6 mice. Mice were injected with 1×10^5^ live MCA-205, MCA-205-OVA, MCA-205-E1A-Δp300-OVA, or MCA-205-E1A-OVA tumor cells s.c. in the flank three times, five days apart. Five days after last tumor dose (day 20), mice were challenged with serial log dilutions of MCA-205-OVA tumor cells on the contralateral flank from 1×10^1^–1×10^6^ cells. The TPD_50_ of MCA-205-OVA was calculated six weeks later. Data shown is the mean ± SEM from three experiments, with three mice per dose per experiment. ***, p<.001.

The observation that MCA-205-OVA and MCA-205-E1A-Δp300-OVA cells were equally tumorigenic as parental MCA-205 cells (Figure 2) despite their capacity to elicit an OVA-specific T cell response was puzzling. One possible explanation for these results was that MCA-205-OVA and MCA-205-E1A-Δp300-OVA tumors suppressed OVA-specific T cells in the tumor microenvironment, but not at sites distal to the primary tumor. If this hypothesis were correct, mice with primary MCA-205-OVA tumors in one flank should be resistant to a subsequent challenge with a tumorigenic dose of MCA-205-OVA cells in the contralateral flank. Therefore, we injected B6 mice with either PBS, MCA-205-OVA, MCA-205-E1A-Δp300-OVA or MCA-205-E1A-OVA tumor cells into the flank of mice, on three occasions, and then determined the TPD_50_ of MCA-205-OVA tumor cells injected on the contralateral flank (Figure 5). As predicted, mice that received injections of PBS did not exhibit measurable anti-tumor immunity to a subsequent challenge with MCA-205-OVA cells. In contrast, immunization of B6 mice with tumorigenic MCA-205-OVA cells and MCA-205-E1A-Δp300-OVA cells, as well as non-tumorigenic MCA-205-E1A-OVA cells, all induced significant protection against subsequent challenge with tumorigenic doses of MCA-205-OVA cells (Figure 5). Following injection of MCA-205-OVA, MCA-205-E1A-Δp300-OVA, or MCA-205-E1A-OVA tumor cells, there was an approximately 1,000 fold increase in the numbers of MCA-205-OVA cells required to form tumors on the contralateral flank compared to PBS primed mice (Figure 5). Concomitant tumor immunity occurred even in the presence of growing tumors from the primary inoculation of MCA-205-OVA and MCA-205-E1A-Δp300-OVA cells.

**Figure 6:**
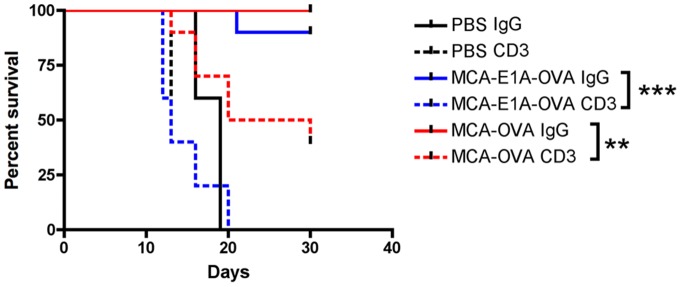
Concomitant tumor immunity induced by administration of MCA-205-E1A-OVA or MCA-205-OVA tumor cells requires T cells. Mice were injected with PBS or 1×10^5^ live MCA-205-OVA or MCA-205-E1A-OVA tumor cells s.c. in the flank three times, five days apart. Five days after last tumor dose (day 20), mice were challenged with 1×10^4^ MCA-205-OVA tumor cells on the contralateral flank. Mice were administered two injections of 10 μg of anti-CD3 or control IgG before and after MCA-205-OVA tumor challenge on days 17, 19, 22 and 24. Survival curves show the percentage of mice without tumor from MCA-205-OVA cell challenge only. Data was analyzed by the log-rank (Mantel Cox) test. **, p<.01; ***, p<.001.

Next, we determined if T cells were required for concomitant tumor immunity induced by MCA-205-OVA and MCA-205-E1A-OVA cells. Mice were inoculated with PBS, MCA-205-OVA or MCA-205-E1A-OVA cells three times and then T cells were depleted with CD3 antibody 2C11 [19] on days 17, 19, 22 and 24. Mice were subsequently challenged with MCA-205-OVA tumor cells on the contralateral flank on day 20. Following MCA-205-OVA tumor cell challenge on the contralateral flank, we determined the percentage of mice which rejected MCA-205-OVA tumor cell challenge. These results showed that mice primed with either MCA-205-OVA tumor cells or MCA-205-E1A-OVA tumor cells and depleted of T cells were markedly impaired in their ability protect against challenge by tumorigenic doses MCA-205-OVA cells on the contralateral flank. Control IgG antibody had no effect on the induction of concomitant tumor immunity (Figure 6). Compared to mice primed with MCA-205-E1A-OVA cells and T cell depleted, mice primed with MCA-205-OVA cells and T cell depleted had fewer MCA-205-OVA tumors with subsequent challenge. The reason for this discrepancy is unclear.

### TAMs from MCA-205-E1A-OVA tumors express significantly less arginase than TAMs from MCA-205-OVA tumors

**Figure 7:**
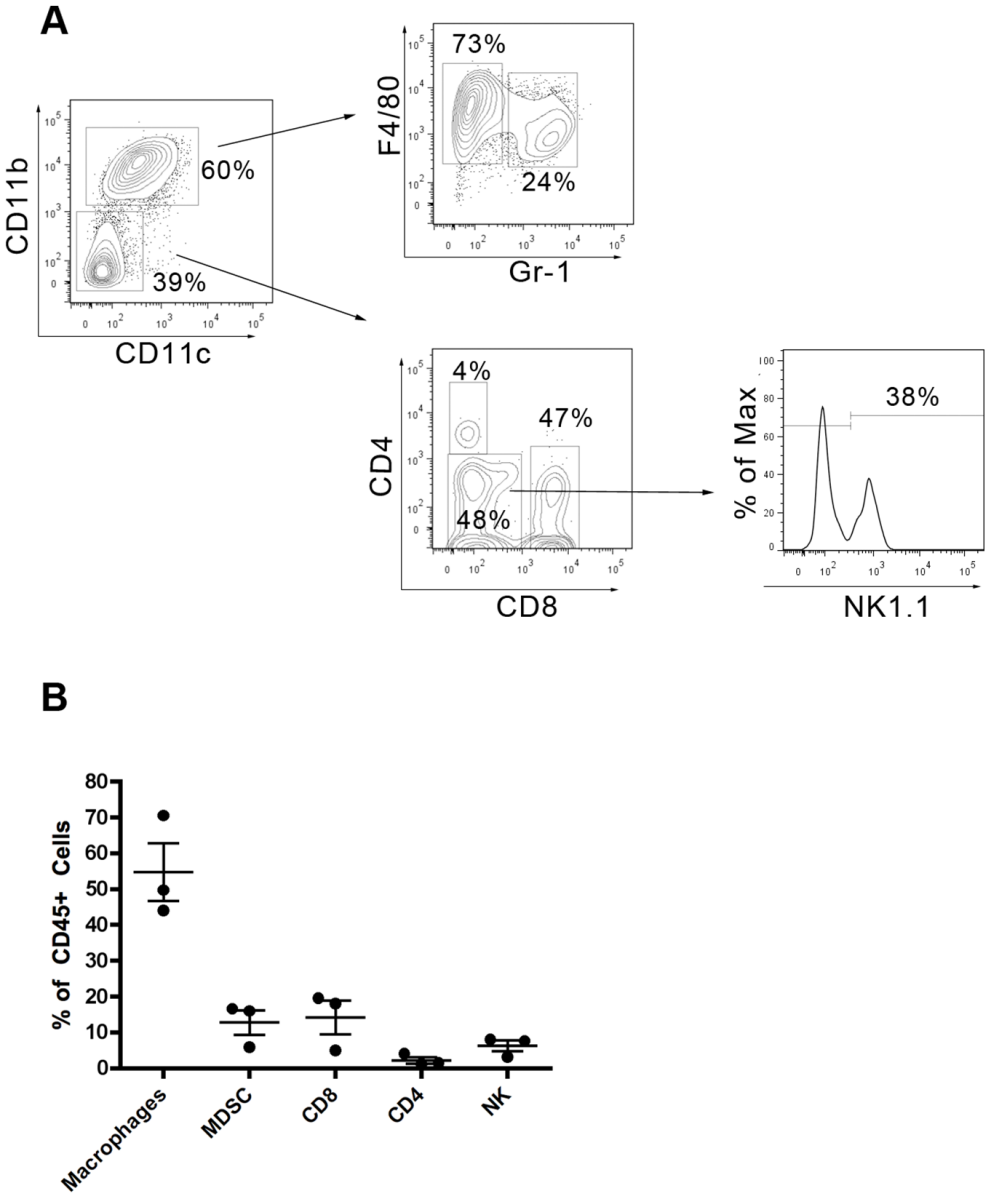
Composition of immune infiltrate of MCA-205-OVA tumors. **A**) Representative gating strategy to determine immune infiltrate of MCA-205-OVA tumors. B6 mice were administered 1×10^5^ MCA-205-OVA cells, and tumors were excised and digested when the tumor reached 15 mm. The immune cells which infiltrated the tumor were determined by flow cytometry. CD45^+^, live cells were first gated. Macrophages (CD45^+^CD11b^+^ CD11c^lo^ F4/80^+^GR-1^−^), myeloid-derived suppressor cells [(MDSC) CD45^+^CD11b^+^ CD11c^lo^ GR-1^+^], CD4 T cells (CD45^+^CD11b^−^CD3^+^CD4^+^), CD8 T cells (CD45^+^CD11b^−^CD3^+^CD8^+^), and NK cells (CD45^+^CD11b^−^CD3^−^CD8^−^CD4^−^NK1.1^+^) were quantified in **B** as the percentage of CD45^+^ cells. Tregs were <1%, not shown in figure. Data shown is the mean ± SEM from three mice.

Our data suggests that the systemic anti-tumor T cell response was similar following challenge with tumorigenic MCA-205-OVA cells and non-tumorigenic MCA-205-E1A-OVA cells. Therefore, we investigated the MCA-205-OVA tumor microenvironment at the primary site of inoculation in an effort to determine why these mice failed to reject primary MCA-205-OVA tumors. We first quantitated the types of immune cells (macrophages, CD4 and CD8 T cells, NK cells and myeloid-derived suppressor cells) present in the MCA-205-OVA tumors by flow cytometry (Figure 7). We found that macrophages were the predominant inflammatory cell in MCA-205-OVA tumors contributing to nearly 55% of the CD45^+^ cells; this was nearly four-fold higher than CD8 T cells, the next most frequent immune cell in tumors (Figure 7). CD4 T cells (2%) and regulatory T cells (<1%) were notable for their relative absence in progressive MCA-205-OVA tumors.

**Figure 8:**
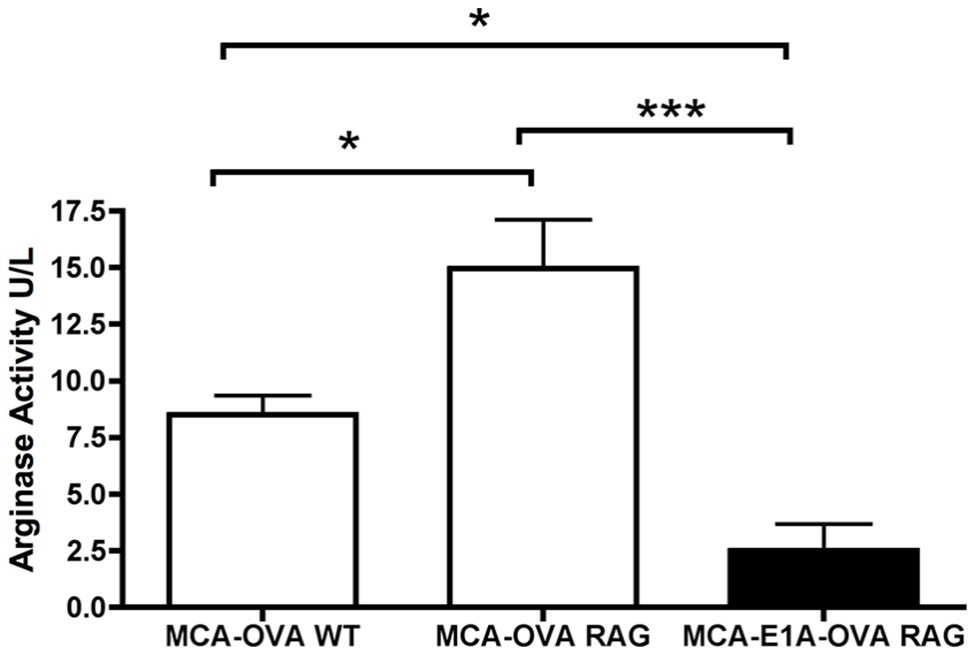
TAMs from MCA-OVA tumors have significantly more arginase activity compared to TAMs from MCA-E1A-OVA tumors. RAG deficient mice were administered 1×10^6^ MCA-205-OVA or MCA-205-E1A-OVA tumor cells, and WT mice were given 1×10^6^ MCA-205-OVA tumor cells. After tumors reached approximately 15 mm, tumors were excised and digested, and CD45^+^CD11b^+^ F4/80^+^GR-1^−^ macrophages were purified by FACS and tested for arginase-1 activity at a concentration of 1×10^5^ TAMs per 100 μL of lysis buffer using a colorimetric assay. Data shown is the mean ± SEM from four-six mice per group. Data were analyzed by ANOVA followed by Tukey's HSD post hoc analyses. *, p<.05 ***, p<.001.

Tumor associated macrophages (TAMs) are known to be pro-tumorigenic and known to actively suppress the immune system through the production of suppressive immune mediators [20]–[22]. One major mechanism whereby TAMs suppress anti-tumor immune responses is through the production of arginase-1. Arginase-1 expression by TAMs has been associated with the suppressive tumor microenvironment and shown to inhibit anti-tumor T cell responses [23]–[25]. Importantly, arginase-1 is usually only suppressive in the tumor microenvironment locally and does not lead to systemic immunosuppression. Because our data showed that the systemic anti-OVA T cell response in mice with MCA-205-OVA tumors was intact, arginase-1 production by TAMs was investigated. To compare arginase-1 expression between TAMs from MCA-205 or MCA-205-E1A tumors, we grew MCA-205-OVA tumors in B6 or B6 RAG^−/−^ mice and purified TAMs by FACS. MCA-205-E1A-OVA cells are non-tumorigenic in WT B6 mice; therefore, RAG^−/−^ mice were used to generate MCA-205-E1A-OVA tumors. Lysates from the different TAMs were analyzed for arginase-1 activity (Materials and Methods). These results showed that TAMs from MCA-205-OVA tumors grown in both WT and RAG^−/−^ mice produced large amounts of arginase-1 (Figure 8). Similar results were obtained with MCA-205 tumor cells (data not shown). In contrast, TAMs from MCA-205-E1A-OVA tumors produced negligible amounts of arginase-1 in RAG^−/−^ mice.

**Figure 9:**
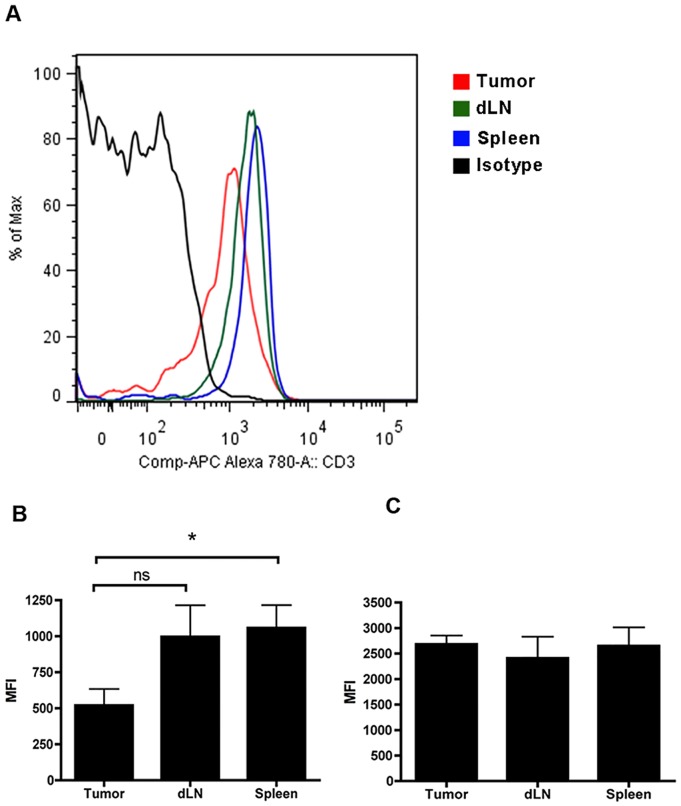
Surface expression of CD3ε on MCA-205-OVA tumor infiltrating CD8 T cells is reduced compared to CD8 T cells from spleens of tumor bearing mice. **A, B**) B6 mice were injected with MCA-205-OVA tumor cells and tumors were excised and digested once they reached a diameter of 15 mm. The expression levels of surface CD3ε on CD8 T cells from tumor, draining lymph node (dLN) or spleen of tumor bearing mice was determined by flow cytometry. **A**) A representative histogram of CD3ε expression is shown. **B, C**) MFI of CD3ε expression on CD8 T cells (**B**) or CD4 T cells (**C**) from either tumor, draining lymph node (dLN) or spleen of mice with MCA-205-OVA tumors. Data shown is the mean ± SEM from four mice for tumor and spleen, and three mice for draining lymph node. Data were analyzed by ANOVA followed by Tukey's HSD post hoc analyses. *, p<.05; ns, no significance.

High arginase expression by TAMs is associated with low L-arginine levels within the tumor. L-arginine is a critical amino acid for T cells, and one of the effects of low arginine levels on T cells is the reduction of CD3ε on the cell surface [25]. Therefore, we compared surface expression of CD3ε on T cells found in the tumor, the draining lymph node and the spleen of MCA-205-OVA tumor bearing mice. Our results show that CD8 T cells from MCA-OVA tumors expressed significantly less surface CD3ε than CD8 T cells from the spleen, but were not significantly different than CD8 T cells from the draining lymph node (Figure 9 A, B). CD4 T cells exhibited no change in the surface expression of CD3ε between the tumor, draining lymph node and spleen (Figure 9 C). Collectively, these data are consistent with the hypothesis that arginase-1 producing TAMs present in the tumor microenvironment induce local, but not systemic, suppression of anti-tumor immunity following injection of MCA-205-OVA tumor cells. In contrast, E1A expression in MCA-205 cells inhibits arginase-1 expression by TAMs allowing for an effective local anti-tumor immune response.

## Discussion

In this study we used OVA as a model tumor antigen to evaluate the ability of E1A expression to augment antigen-specific anti-tumor T cell responses in mice. We found that the expression of an E1A-OVA fusion protein rendered MCA-205 cells essentially non-tumorigenic in normal B6 mice. In contrast, the tumorigenicity of MCA-205 cells was not substantially changed by the expression of OVA or E1A-Δp300-OVA in normal B6 mice. Immunization with either MCA-205-OVA, MCA-205-E1A-Δp300-OVA, or MCA-205-E1A-OVA tumor cells induced a robust OVA-specific anti-tumor T cell response. For example, following injection of either MCA-205-OVA, MCA-205-E1A-Δp300-OVA, or MCA-205-E1A-OVA tumor cells, approximately 1,000 fold more MCA-205-OVA cells were required to form tumors in comparison to tumor challenge with naïve mice (Figure 5). These results indicated that while MCA-205-OVA, MCA-205-E1A-Δp300-OVA induced systemic immunity, local tumor immunity was hampered as progressive tumors formed at the site of the primary challenge site.

Based on the observation of concomitant tumor immunity in the presence of primary tumor formation, we investigated the tumor microenvironment. We found that TAMs were the predominant inflammatory cell in progressive MCA-205-OVA tumors in WT B6 mice. TAMs isolated from MCA-205-OVA tumors, in either WT B6 mice or B6 RAG−/− mice, expressed large amounts of arginase-1. In contrast, TAMs from MCA-205-E1A-OVA tumors from B6 RAG−/− mice expressed negligible amounts of arginase-1. TAMs are often phenotypically similar to alternatively activated macrophages, producing high levels of arginase-1, which depletes the local environment of L-arginine [26]. T cells in an environment depleted of L-arginine express lower levels of CD3ε on the cell surface, reduced total levels of CD3ζ, become arrested in the G_0_–G_1_ growth phase and display a decrease in global protein translation [25], [27]. We found that CD8 T cells which had infiltrated MCA-205-OVA tumors had significantly lower surface CD3ε than CD8 T cells from the spleen of MCA-205-OVA tumor bearing mice, a finding that is consistent with high arginase-1 activity. Collectively, our results are consistent with the hypothesis that E1A expression in tumors may preserve antigen-specific anti-tumor T cell responses in the local tumor environment by inhibiting the production of arginase-1 by TAMs, a biological activity of E1A not previously described. Alterations in arginase-1 activity in the tumor microenviroment also provide a possible explanation for concomitant tumor immunity induced by MCA-205-OVA tumors in the presence of a progressive primary MCA-205-OVA tumor.

Future studies need to directly examine the role of arginase-1 production by TAMs, concomitant tumor immunity and the anti-tumorigenic effect of E1A. Definitive studies examining the role of arginase-1 production by TAMs and anti-tumor immunity are currently hampered by a relative lack of arginase-1 specific reagents. The most commonly used in inhibitor of arginase is N(ω)-hydroxy-nor-L-arginine (nor-NOHA). Nor-NOHA inhibits both arginase-1 and arginase-2 and in conventionally used doses the inhibition of arginase-1 *in vivo* is less than 50% [28]. Mice genetically deficient in arginase-1 [29] or conditionally-induced to be deficient in arginase-1 [30], succumb to an illness that mimics human arginase deficiency far too rapidly to perform tumor induction experiments examining concomitant tumor immunity. The development of mice conditionally deficient of arginase-1 in macrophages or better arginase-1 inhibitors will facilitate progress in this important area.

The mechanism whereby tumor cells that express E1A lead to the decreased expression of arginase-1 by TAMs is unknown and also needs to be explored. Arginase-1 activity has been postulated as one of the mechanisms for the failure of adoptive cellular immune therapy to be effective against solid tumors. Determining how E1A is able to inhibit tumor cells from inducing arginase-1 in TAMs could have important implications in augmenting local anti-tumor immune responses in the setting of progressive tumor enlargement. Additionally, novel uses of E1A could be considered to augment local anti-tumor immune responses. For example, studies could be performed to determine if administration of liposomes with E1A protein into a tumor could augment local tumor anti-immune responses and lead to tumor rejection.

Finally, the use of the MCA-205-OVA, MCA-205-E1A-Δp300-OVA, or MCA-205-E1A-OVA tumor lines in the B6 mouse is a new model of concomitant tumor immunity that also allows for the quantitation of antigen-specific T cell responses. In the B16 melanoma concomitant tumor immunity model developed by Turk *et. al*.[31], concomitant tumor immunity was observed only after manipulating B16 melanoma cells to express GM-CSF, or by depleting/inhibiting regulatory T cells. In our MCA-205-OVA model, concomitant tumor immunity occurred by day 20, and required no further experimental manipulation of the tumor cells or B6 mice. To our knowledge, this model of concomitant tumor immunity is unique and may more accurately replicate ongoing systemic anti-tumor immune responses in a human in the face of enlarging primary or secondary tumors.
