# Correction: Possible Role of Arginase-1 in Concomitant Tumor Immunity

**DOI:** 10.1371/journal.pone.0153122

**Published:** 2016-03-31

**Authors:** Michael J. Korrer, Yuwen Zhang, John M. Routes

Dr. Yuwen Zhang should be included in the author byline. Dr. Zhang should be listed as the second author and is affiliated with #1–5: 1 Department of Pediatrics, Medical College of Wisconsin, Milwaukee, Wisconsin, United States of America; 2 Department of Microbiology and Molecular Genetics, Medical College of Wisconsin, Milwaukee, Wisconsin, United States of America; 3 Children's Research Institute, Milwaukee, Wisconsin, United States of America; 4 Cancer Center, Medical College of Wisconsin, Milwaukee, Wisconsin, United States of America; 5 Department of Microbiology and Immunology, University of Louisville, Louisville, KY 40202. The contributions of this author are as follow: Performed the experiments and analyzed the data.

The correct citation is: Korrer MJ, Zhang Y, Routes JM (2014) Possible Role of Arginase-1 in Concomitant Tumor Immunity. PLoS ONE 9(3): e91370. doi:10.1371/journal.pone.0091370
